# To neutrally offer or strongly recommend? General practitioners’ perspectives on screening for gestational diabetes according to the national guideline in Norway

**DOI:** 10.1080/02813432.2024.2378204

**Published:** 2024-07-15

**Authors:** Ingeborg Forthun, Kathy Ainul Møen, Stefán Hjörleifsson

**Affiliations:** a Department of Global Public Health and Primary Care, University of Bergen, Bergen, Norway; b Department of Disease Burden, Norwegian Institute of Public Health, Bergen, Norway; c Research Unit for General Practice Bergen, NORCE Norwegian Research Centre, Bergen, Norway

**Keywords:** primary care, Norway, gestational diabetes, guideline, General practitioner

## Abstract

**Objective:**

To explore general practitioners’ experiences and reflections on how the current Norwegian guideline for screening for gestational diabetes affects their clinical practice.

**Design:**

A qualitive study in which data were collected through semi-structured focus group interviews and analyzed thematically.

**Setting and subjects:**

Five focus groups conducted in 2020 among GPs in Norway; three interviews took place face-to-face and two were held digitally. The total number of participants was 31.

**Results:**

GPs acknowledged the potential benefits of more extensive screening, but had concerns about the medicalization of pregnancy, stating that some women experienced considerable anxiety. The GPs expressed doubts about the guideline’s evidence base but differed in how they interpreted what the guideline was asking them to do. Some offered eligible women the opportunity to be screened, while other set up a screening appointment without consulting the women first. For some, fear of incrimination made them recommend screening without being convinced that it was the right thing for the patient.

**Conclusions:**

It is unclear whether the guideline for gestational diabetes requires GPs to recommend screening to pregnant women or if they should provide neutral information about the availability of screening. This ambiguity should be addressed, and the guideline evaluated against the core principles of general practice.

## KEY POINTS

The Norwegian guideline for gestational diabetes (2017) increased the proportion of pregnant women eligible for screening and spurred considerable debate.

- The GPs in this study expressed concerns about the lack of evidence and the consequences of screening more women for gestational diabetes.
- It is unclear to GPs whether they are required to recommend screening to pregnant women or just inform them of its availability.
- The guideline should be evaluated to better account for the benefits, burdens and costs of screening and any subsequent treatment.

## Introduction

Currently, there is no international consensus on either the diagnostic or the inclusion criteria for screening for gestational diabetes mellitus (GDM), and the evidence on the effects of screening based on differing criteria is insufficient [1–3]. In 2013, the World Health Organization (WHO) published new diagnostic criteria and definitions of diabetes mellitus in pregnancy and GDM [4]. Based on the new recommendations from WHO and new epidemiological evidence on causes, consequences, and effect of treatment, the Norwegian Directorate of Health published a new guideline for GDM in April 2017 [5]. The Directorate argued that a change in guideline was needed due to the increasing proportion of pregnant women with high BMI and/or increased age in Norway, and the fact that approximately 50% of women with GDM were undiagnosed when the old criteria were used.

The new Norwegian guideline introduced in 2017 recommends screening for GDM in weeks 24-28 of pregnancy for women who fulfill one or more of a fixed set of criteria. Screening is by measuring fasting plasma glucose followed by a 2-h 75 g oral glucose tolerance test (OGTT) [5]. The screening policy was changed from the previous guideline following a systematic review of the evidence using the GRADE framework [6]. Most notably, the criteria for GDM screening based on BMI and age changed: from pre-pregnancy BMI > 27 in the old guideline to >25 in the new one; and from >38 years to >25 years for primiparous, and >40 for multiparous (all women). Under the previous guideline, approximately 25% of pregnant women were screened for GDM, while under the new guideline it was estimated that approximately 70% of pregnant women would be screened [5]. If followed, this implies that 25 000 more pregnant women are screened for GDM every year in Norway.

A nationwide registry-based study that estimated the prevalence of GDM in Norway in the period before and after the new guideline was implemented found that the prevalence of GDM increased from 2010 to 2016 and then plateaued [7]. The proportion of women diagnosed with GDM at the recommended time of screening and who received pharmaceutical treatment, however, increased substantially following the implementation of the guideline in 2017. This study did not have data on the proportion of women screened. A recent, small, retrospective study including women who gave birth at the University Hospital of North Norway, found that the proportion of women eligible for screening increased from 46% to 68% [8]. Yet, only 49% of the eligible women were screened. A systematic review and meta-analysis of studies investigating the performance of different screening criteria for identifying GDM found that regardless of the criteria used a large proportion of pregnant women would have to be screened to identify most women with GDM [9]. There is, however, no clear evidence that the identification of more women with GDM results in significant improvements on maternal and infant health [8,10].

The new guideline prompted considerable debate in Norway. Critics, including the Norwegian Association for General Practice [11], raised concerns that screening all primiparous women over 25 years old and lowering the BMI cut-off from 27 to 25 would result in overdiagnosis with a large number of healthy pregnant women being labelled as suffering from a disease [12–14]. This echoed the critique that had been raised internationally following the release of the new WHO definition of GDM that there was insufficient evidence on benefits and possible harms [15]. Those in support of the new guideline argued that 30-50% of women with GDM had not been identified using the old guideline [16,17]. Similar debates on the potential benefits and harms of more extensive screening have been seen in several other countries that have implemented new guidelines for GDM [10,18,19].

Among GPs in Norway and other countries, the debate on screening for GDM can be viewed as a reflection of the ongoing scrutiny of overuse in health care [20,21] and the burdens imposed on patients and healthcare systems [22]. This concern has intensified as a result of evidence demonstrating that well-intentioned guidelines have the potential to overwhelm services [23]. It has also coincide with the publication of the core values and principles of general practice by the Nordic Federation of General Practice [24]. The Federation states that the role of GPs is to provide person-centered care, timely diagnosis and prevention of disease, prioritizing those whose needs for healthcare are greatest, based on the best evidence available, while avoiding unnecessary tests and overtreatment. There is robust evidence that primary health care based on these principles gives good health outcomes and is a global prerequisite for well-functioning health care services [25]. Our own professional backgrounds are from general practice and epidemiology, including epidemiological studies of maternity care. As academics and practitioners, we share a commitment to high quality maternity care, and the last author has long been involved in research about medical overuse.

Despite the debate about increasing the proportion of pregnant women to be screened for GDM, the real-life effects of the new guideline in Norway on GPs’ practice have not been investigated. We therefore conducted this study to explore GPs’ experiences and reflections on how the current Norwegian guideline for screening for GDM affects their clinical practice.

## Material and methods

We conducted a qualitative study with five focus groups. Focus groups were chosen to elicit rich accounts of GPs’ experiences with the guideline through their interaction in the group. The scope of the research objective was broad, and together with our use of thematic analysis and sampling approach that was partly based on convenience, this indicated that a rather large sample size might be needed. On the other hand, the study is theoretically founded (i.e. on the core values of general practice), and the interviews were conducted by experienced researchers in the field (KAM and SH) with the skills to establish good dialogue. Based on these considerations, we judged five groups to give sufficient information power [26] for the purpose of our study. Information power and sample size were assessed both before recruitment and after conducting the first three interviews.

### Participant selection and recruitment

GPs were contacted by e-mail through KAM and SH’s professional network and by contacting the supervisors of GPs’ continuing education group meetings as we wanted to elicit the experience of GPs of varied experience and age. Of the five focus groups, four included GPs within the same GP preactice, while the last group comprised a compulsory education meeting for GP trainees (the supervisor did not take part in the focus group).

There were a total of 31 participants: 19 women and 12 men. Their age and length of experience as GPs varied, and they practiced in rural and urban areas in different parts of Norway. The number of participants, age range and years working as a GP are given in Table 1.

**Table 1.** Characteristics of participants in the focus groups.

Focus group no	N	Age in years	Experience as GP in years	Female
1	4	32–63	1–30	2
2	3	34–54	2.5–26	2
3	3	34–43	4–10	2
4^1^	10	29–66	0.25–37	6
5^1,2^	11	30–40	3–5	7

^1^ Conducted digitally.

^2^ Compulsory continuing education meeting.

Due to COVID-19 restrictions, two of the five focus groups took place digitally. All five focus groups were conducted between October and November 2020.

### Data collection and analysis

A semi-structured interview guide was developed based on previous studies and discussion among the authors. One pilot interview was conducted to refine the interview technique and adjust the interview guide. The final interview guide (Supplementary Appendix 1) comprised one open question with several follow-up questions to expand the responses. The interviews were conducted by two of the authors (either KAM and IF, or SH and IF). Before attending the focus group, each participant was asked to review the three or four most recent pregnancies they had followed, particularly regarding GDM screening. At the beginning of each focus group, the interview topic was briefly introduced by going through the inclusion criteria for screening for undiagnosed diabetes and GDM in the national guideline. We then asked the participants to discuss their experiences with screening women for GDM. The interviews lasted for 60 to 90 min, were audio recorded, anonymized, and then transcribed verbatim by two medical students.

We analyzed the interviews using systematic text condensation [27], a method for thematic cross-case analysis of qualitative data well suited for focus groups. Our analysis took the following steps: (i) read the material to gain an overview and elicit preliminary themes; (ii) develop code groups from preliminary themes; (iii) establish subgroups exemplifying vital aspects of every code group by condensing the content of each and identifying illustrating quotes and (iv) synthesize the condensates. The transcripts were read through several times by all three authors, and preliminary themes and code groups were suggested and discussed. Each author was assigned a specific theme, continued to develop code groups for that theme, condensed the content and identified quotes for each group. These suggestions were discussed back and forth in several meetings before the authors agreed on the final version.

In developing the interview guide, during data collection and data analysis we were conscious of our preconceptions and positions, and continuously discussed how these could affect our methodological choices, recruitment of participants, and the interpretation of data. In our meetings, we thus mitigated the risk of potential biases by ensuring adherence to our study objective and reflecting strictly on the experiences of the GPs in the focus groups.

### Ethical approval

Prior to the focus groups, the GPs received written and oral information on the study and signed a consent form. All personal information was handled according to the General Data Protection Regulation. The project was assessed by the Regional Committee for Medical and Health Research Ethics (ref. 176974] and evaluated as exempt from review. As the project did not collect any sensitive personal data, we were not required to notify the Norwegian Centre for Research Data (NSD).

## Results

The GPs were aware of the new guideline for screening for GDM and eagerly shared their experiences and opinions. The findings from the interviews were organized into five main themes. First, the GPs described how the new guideline presented an opportunity to identify more women with GDM and initiate conversations on lifestyle. Second, screening posed logistical challenges and subsequent treatment and follow-up of women with GDM was viewed as resource intensive. Third, the GPs were concerned that screening more women and lowering the threshold for GDM contributed to overdiagnosis and overtreatment. Fourth, they were not convinced that the higher prevalence of identified cases had any effect on complications and requested more evidence and an evaluation of benefits and harms. And fifth, the GPs differed in their interpretation of what the guideline was asking them to do.

### Theme 1: an opportunity for dialogue on lifestyle

While some GPs had not identified more women with GDM since the introduction of the new guideline, others had identified additional cases. Among these were women they had not expected to have GDM based on clinical assessment, and women whom they suspected might have had undiagnosed diabetes in a previous pregnancy (large baby, glucose in urine). Two GPs described how screening had provided a starting point for a constructive dialogue with pregnant women who were overweight or who led an unhealthy lifestyle:

An oral glucose tolerance test when a patient has high BMI, it’s really a nice way to talk about BMI with pregnant women. Because this is a sensitive topic, and maybe in particular when they are pregnant, so I think this has been a good way to initiate that conversation. (Martha, GR2)

Among the additional cases of women diagnosed with GDM, a large proportion had OGTT values just above the cut-off, and dietary advice was often sufficient to achieve blood glucose levels in the normal range. Another potential benefit the GPs noted was the opportunity to inform women of their increased risk of type 2 diabetes.

### Theme 2: Resource intensive screening and follow-up

The GPs were not heavily involved in the screening itself and it accounted for a low proportion of their own workload, but they still saw it as a resource intensive task for the GP practices. For large practices in particular, the increased number of pregnant women eligible for screening could pose logistical challenges. GPs were required to reorganize their schedules and find suitable spaces for women to sit in for two hours after drinking the glucose solution. As one GP explained:

It is, after all, a resource-intensive thing, the oral glucose tolerance test. On some occasions, there have been pregnant women in every room. We are a large GP center where we have rooms which we use as emergency room, for wound treatment and for different procedures, and occasionally there are pregnant women who sit in the corridors. (Oscar, GR4)

The GPs themselves were involved in the follow-up of women who were diagnosed with GDM. These women often had to be trained in how to measure their glucose. Regular measurement and monitoring were time-consuming for the women. Follow-up required additional consultations with GPs, and sometimes, if the treatment target was not met, referral to specialist care.

### Theme 3: Anxiety, worries and a barrier to person-centered care

Several of the GPs expressed concern that broader screening criteria in the new guideline changed how a pregnancy was viewed from a natural, healthy state to being at risk of disease. A lower threshold for GDM meant that many hitherto healthy pregnant women were labelled as diseased. The GPs had observed that fear of complications could cause anxiety in these women, and that the diagnosis could result in shame and hurt. One GP shared stories of two patients whose pregnancies had been uncomplicated until they were diagnosed with GDM. The diagnosis caused a great deal of anxiety and worry and affected the rest of the pregnancy. This made the GP question whether the diagnosis had done more harm than good.

It is hard to watch how a pregnancy, that in so many ways is something very positive, becomes such a big source of worry that they are on sickness leave throughout the whole pregnancy because they are worried, and not because of the diabetes in itself. (Lisa, GR 1)

Another negative consequence of the guideline was that it sometimes got in the way of patient-centered care. Being preoccupied with the tasks required by the guideline, the GP could be less responsive to the patient’s individual needs. As a result, the patient could end up getting worse care:

It fills up your time so there is less time for what I would rather like to prioritize. You become so preoccupied with following the guideline and avoiding malpractice so I can’t … one forgets what the patient … that the patient perhaps has an individual need. (Peter GR 1)

### Theme 4: Doubts about the evidence-base

The GPs felt that they did a lot of screening without really detecting much, and questioned whether it was important to identify these mild cases of GDM:

It is nice to detect and help prevent complications, I am just wondering how many more complications there would have been? (Caroline, GR 5)

The GPs who had experienced worries and anxiety among women with GDM were concerned that the effect of screening more women on their mental health had not been accounted for when the guideline was developed. While they acknowledged that screening inevitably comes at a certain cost and that a certain amount of concern could be constructive in, for example, making a lifestyle change, they were not convinced that the benefits outweighed the harms:

But to be worried and sad and desperate about the situation, it is ok if it is useful. Because everyone wants to do this if it really matters for their unborn child. So, what we must know is, kind of, is it worth it? (Ana, GR1)

The GPs repeatedly voiced doubts about the evidence base for the current guideline and questioned the cost-effectiveness of screening and the overall balance of benefits and harms. In particular, the age criterion in the new guideline (>25 years old for primiparous women) was seen as problematic since it meant that a very high percentage of primiparous women were eligible for screening – women who often did not fulfill any of the other criteria. One of the GPs also stated that it would have been easier to accept the new guideline if there had been a plan to evaluate it from the outset.

When this guideline was implemented, you would expect that an evaluation should be conducted and that there then is a conclusion about whether the guideline should be continued. Whether there is anything to gain. (Christian, GR3)

### Theme 5: Handling the responsibilities outlined in the guideline

The GPs’ interpretation of what it meant to follow the guideline and the burden of responsibility it placed on themselves and on the pregnant women in their care differed strongly. In one of the focus groups the ambiguous meaning of the concepts “recommend” and “offer” in the guideline was discussed at some length:

Christian: I was looking at the wording in the recommendations. It says to “offer glucose tolerance testing”. It does not say that we are required to recommend the testing. It means we are supposed to [say] “if you want to, then I can offer you a glucose tolerance test”.

Sara: But there is a strong recommendation that you offer the test.

Christian: Yes, but what I am supposed to do is to offer it to the patient.

Sara: But according to the guideline, there is a weak recommendation that we should offer HbA1C testing.

Christian: Does that mean that I should not make a strong recommendation to the patient about this? (GR 3)

Thus, one position was that the guideline requires the GP to make eligible pregnant women aware that they can have an OGTT, but it does not instruct them to recommend that the patient accepts this offer. Another position was to interpret the guideline as requiring the GP to recommend that the pregnant women should have the OGTT, i.e. “Unless they are very young or very thin, they should have an OGTT. That is just how it is” (Jacob, GR 4) or even to “just set up the patients for the test” (Aina GR 4) without giving the women a chance to decide whether this was something they wanted or not. In the latter position, the GPs would argue that the national health authorities had decided that pregnant women should be screened and that it was not up to the individual GP to question this decision. The experiences and perspectives of the participating GPs seem to indicate a complex mixture of these two positions. The following quote demonstrates how the GP can take both positions during a single interaction with a patient. The GP sets out telling the patient that the guideline *requires* her to take the test but then moderates himself to explain that the test is something that is *offered* to the woman and that she has a choice:

‘You are 26 years old, so you should take an oral glucose tolerance test, according to the guideline. If you do not want it, then I need to write down in your patient record that you are actively deviating and that I cannot be blamed.’ […] In a way, you tell the patient that it is her own fault if she does not take the test [….], ‘we offer this test based on the guideline, but you have, you have a choice, this is not compulsory’. It’s a bit of a weird dynamic in a way. (Simon, GR1)

This quote also illustrates some of the GPs’ contradictory thoughts about where the duties imposed by the guideline might be placed. Regardless of the position taken, the GPs were eager to avoid incrimination if an eligible woman was not screened. The fear of incrimination and the corresponding need to cover one’s own back could even lead the GPs to strongly recommend that pregnant women should have an OGTT, even if they were not convinced this was the right thing for the patient.

## Discussion

### Main findings

The GPs in this study described how the change in screening criteria in the guideline for GDM from 2017 could lead to the identification of more women with GDM, foster constructive dialogues regarding lifestyle, yet also induce worries and the medicalization of pregnancy. The GPs raised doubts regarding the evidence base and the cost-effectiveness of screening so many women. However, the GPs differed in their interpretation of the guideline, ranging from neutrally offering screening to strongly recommending it to eligible women, or even scheduling screening without consulting the patient. The GPs’ positions seemed to be related to how they weighed the importance of avoiding later incrimination, leading some to recommend screening even if they did not believe that this was in the woman’s best interests. In these instances, the GP would emphasize documenting that they had offered screening, particularly if a woman declined.

### Strengths and limitations

We have elicited rich accounts of GPs’ experiences and opinions regarding a complex and important issue for maternity care in Norway and globally. Previous studies on guidelines for GDM have focused mostly on implementation, awareness of guidelines among physicians, compliance, and cost-effectiveness [8,10,15, 28–32], but few have explored GPs’ perspectives [33]. Our sample included GPs of both sexes and of varied age and experience, and we believe that we have sufficient information power to state that the results are relevant to the uptake and consequences of the current guideline for GDM in Norway.

The introduction of the guideline spurred considerable debate in Norway, and the Norwegian Association of General Practice was one of its critics [11]. The debate was not brought up by the authors during recruitment or data collection, but it was evident in the focus groups that the GPs were aware of it. Expression of strong attitudes against screening in the focus groups may have prevented some participants from expressing other views and it is possible that some participants may have been hesitant to express or elaborate their opinions. However, the abundance of tensions in the current data material (e.g. benefits vs. harms and contrasting interpretations of what the guideline requires the GP to do), indicates that the participants probably felt free to offer their sincere opinions. Other data sources, including individual interviews might have yielded complementary results. Further, two of the focus groups were held digitally because of the Covid-19 pandemic. The verbal and social interaction in digital interviews are somewhat different than when participants and researchers are in the same room, and the conversation among participants in the digital forum was less fluent. The limiting effects of the digital setting were, however, mitigated by the participants knowing each other. Four of the five group interviews were among GPs in the same practice. While this introduces the possibility that the participants may have influenced each other’s opinions beforehand, the dialogue between individuals who already know each other usually flows more freely and enables shared reflections more easily than dialogue between strangers.

### Findings in relation to other studies

The GPs in this study acknowledged that increasing the proportion of pregnant women who are screened is likely to lead to the identification of more cases of GDM and described situations where screening opened a conversation that would enable the pregnant woman to change her lifestyle for the better. The opportunity this provided for addressing the risk of type 2 diabetes was also highlighted. Lifestyle interventions among women with a history of GDM have shown efficacy in reducing the incidence of diabetes or delaying its onset [34]. Previous research [35–37], including two recent studies from Norway [38,39], clearly demonstrates that patients are positive about GPs guiding them to a more healthy lifestyle. However, although it is a core task for GPs to provide diagnosis and (preventive) treatment or health promotion when indicated [24], this principle must be balanced against the requirement to avoid overdiagnosis, overtreatment and potentially harmful medicalization. The GPs in our study had observed that screening could be burdensome to the pregnant women in the sense that it could cause excessive worries. Similar experiences were reported in a Norwegian qualitative study including 14 women diagnosed with GDM [40], in which the women described feelings of anxiety, guilt and shame when diagnosed, partly due to lack of knowledge and unpredictable implications for the pregnancy. The GPs in the present study expressed concerns about the medicalization of pregnancy and the extensive follow-up and treatment regimens for women found to have GDM. The GPs were thus concerned that the guideline involves screening a too large proportion of pregnant women in Norway and that this leads to harm, a concern that aligns with previously published criticism of the guideline [12–14].

The GPs questioned whether the above-mentioned harm and the additional workload of screening more women for their practice had been accounted for in guideline development. When developing the current guideline, the costs for primary care associated with screening and follow-up of women with GDM were estimated [6]. However, time- and travel costs for the pregnant women and the potential effects of a GDM diagnosis on their mental health was not accounted for. The requirement to consider opportunity costs and the sustainability of the additional workload mandated when issuing a new guideline has recently been highlighted by the Cochrane Network and the Nordic Federation of General Practice. Cochrane states that opportunity costs should be incorporated into evidence considerations [41], and the the Federation’s declaration of core values and principles states that GPs should prioritize those whose needs for healthcare are greatest [24]. This is important because overuse of medical investigations and treatment can cause adverse effects from interventions, psychosocial impacts from labelling, and an excessive treatment burden for patients. Overuse also consumes scarce resources and thus can lead to underuse in other areas, which indirectly causes harm to patients. Therefore, guidelines for generalists, who manage a wide range of conditions and patient groups, need to consider: i) whether sufficient resources are available to expand healthcare; ii) whether the suggested expansion is the best way to use these resources; and iii) if there are other patient groups who have greater unmet needs for health care. The doubts that many of the GPs in our study expressed about the cost-effectiveness of the guideline are borne out by a health economics report mandated by the Norwegian Directorate of Health that was published in 2021 [42]. According to this report, screening pregnant women based on the criteria in the current Norwegian guideline is not cost effective. In the report, the screening criteria BMI ≥ 25 and age > 30 years old were found to be the most cost-efficient but were expected to detect only 42% of cases as opposed to 82% under the current guideline.

Previous research indicates that GPs’ adherence to clinical guidelines is sometimes low [43,44]. As mentioned in the introduction, a recent Norwegian study evaluating the impact of the 2017 guideline for GDM demonstrated low adherence, both before and after guideline implementation [8]. However, the study was small (before group, *n* = 676, after group, *n* = 673) and only included women who gave birth at one hospital. According to a qualitative study with Norwegian GPs, one of the reasons why GPs sometimes deviate from guidelines is that the guidelines may come into conflict with patientcentered care [45]. The results from our study indicate that a fundamental uncertainty about what it entails to adhere to guidelines may also be involved. The guideline recommends that GPs *offer* the test, but there is an ambiguity in the term “offer”: should the GPs just neutrally offer screening, or should they recommend screening? Furthermore, when a patient declines screening, it is uncertain to what extent this can be seen as a failure on behalf of the GP and, consequently, whether such instances can potentially lead to incrimination. In turn, this affects how the guideline is managed. If GPs recommend the OGTT even if they do not believe that this is good for the patient, this can be seen as a diver of medical overuse which has previously been shown in the literature [46,47] namely, that fear of blame and incrimination can lead clinicians to act against what they consider to be the patient’s best interests.

### Implications

The impact and the sustainability of screening for GDM according to the current guideline should be assessed and should evaluate the risk of overdiagnosis, the treatment burden for pregnant women, and the opportunity costs associated with screening and subsequent treatment.

## Conclusions

It is unclear whether the term to “offer” in the current GDM guideline in Norway signifies that GPs should give neutral information or actively recommend screening for eligible women. The wording of the guideline on this point needs assessment, and the guideline should be evaluated against the core principles of general practice with a broad assessment of the benefits, burdens, and opportunity costs of screening and subsequent treatment.

## Supplementary Material

COREQ_checklist.pdf

Appendix 1.pdf
