# Supplementary material for: To neutrally offer or strongly recommend? General practitioners’ perspectives on screening for gestational diabetes according to the national guideline in Norway
Source: Scand J Prim Health Care. 2024 Jul 15;42(4):668–76. doi: 10.1080/02813432.2024.2378204 (PMC11552295; doi:10.1080/02813432.2024.2378204)
Supplement: COREQ_checklist.pdf [file IPRI_A_2378204_SM3798.pdf]

Consolidated criteria for reporting qualitative studies (COREQ): 32-item checklist for manuscript in title: “To neutrally offer or strongly recommend? General practitioners’ perspectives on the national guideline for screening for gestational diabetes in Norway”

No	Item	Guide questions/description	Response
**Domain 1: Research team and reflexivity**			
Personal Characteristics			
1.	Interviewer/facilitator	Which author/s conducted the interview or focus group?	Stated in the methods section.
2.	Credentials	What were the researcher's credentials? *E.g. PhD, MD*	Not stated in the manuscript but see point 3.
3.	Occupation	What was their occupation at the time of the study?	Mentioned in the introduction.
4.	Gender	Was the researcher male or female?	Not explicitly mentioned.
5.	Experience and training	What experience or training did the researcher have?	Mentioned in the method section.
Relationship with participants			
6.	Relationship established	Was a relationship established prior to study commencement?	No.
7.	Participant knowledge of the interviewer	What did the participants know about the researcher? e.*g. personal goals, reasons for doing the research*	At the beginning of each interview, we explained the reasons for doing the research. This is made clear in the methods section and in Appendix 1.
8.	Interviewer characteristics	What characteristics were reported about the interviewer/facilitator? e.g. *Bias, assumptions, reasons and interests in the research topic*	The authors (who conducted the interview) have acknowledged their interests and positions on the research topic in the introduction.
**Domain 2: study design**			
Theoretical framework			
9.	Methodological orientation and Theory	What methodological orientation was stated to underpin the study? *e.g. grounded theory, discourse analysis, ethnography, phenomenology, content analysis*	We have stated that the analytical method was Systematic text condensation which is a version of thematic analysis.
Participant selection			
10.	Sampling	How were participants selected? *e.g. purposive, convenience, consecutive, snowball*	Sampling was part purposive and partly based on convenience. This is stated in the method section.
11.	Method of approach	How were participants approached? e.*g. face-to-face, telephone, mail, email*	A combination of face-to-face and digitally. Stated in the method section.
12.	Sample size	How many participants were in the study?	31 as stated in the method section
13.	Non-participation	How many people refused to participate or dropped out? Reasons?	None.
Setting			
14.	Setting of data collection	Where was the data collected? e.*g. home, clinic, workplace*	Workplace. Stated in the method section.

15.	Presence of non-participants	Was anyone else present besides the participants and researchers?	In one of the groups (the compulsory educational group), the supervisor was present, and we have described this in the manuscript.
16.	Description of sample	What are the important characteristics of the sample? *e.g. demographic data, date*	Table 1
Data collection			
17.	Interview guide	Were questions, prompts, guides provided by the authors? Was it pilot tested?	Yes. This is accounted for in the method section.
18.	Repeat interviews	Were repeat interviews carried out? If yes, how many?	No. This has not been stated explicitly in the manuscript but can be deducted from the method section.
19.	Audio/visual recording	Did the research use audio or visual recording to collect the data?	Yes, audio. Stated in the method section.
20.	Field notes	Were field notes made during and/or after the interview or focus group?	No.
21.	Duration	What was the duration of the interviews or focus group?	60-90 minutes as accounted for in the method section.
22.	Data saturation	Was data saturation discussed?	We have based our sample size considerations on the concept of information power rather than saturation. This is stated and fully accounted for in the method section.
23.	Transcripts returned	Were transcripts returned to participants for comment and/or correction?	No.
**Domain 3: analysis and findings**			
Data analysis			
24.	Number of data coders	How many data coders coded the data?	All three authors coded the data. This is stated in the manuscript.
25.	Description of the coding tree	Did authors provide a description of the coding tree?	All codes consisted of Issues, Themes and Sub-themes.
26.	Derivation of themes	Were themes identified in advance or derived from the data?	They are derived from the data, and this is made clear in the method section.
27.	Software	What software, if applicable, was used to manage the data?	None
28.	Participant checking	Did participants provide feedback on the findings?	No
**Reporting**			
29.	Quotations presented	Were participant quotations presented to illustrate the themes / findings? Was each quotation identified? e*.g. participant number*	Yes.
30.	Data and findings consistent	Was there consistency between the data presented and the findings?	Yes.
31.	Clarity of major themes	Were major themes clearly presented in the findings?	Yes.
32.	Clarity of minor themes	Is there a description of diverse cases or discussion of minor themes?	Some minor themes have been included but the discussion focuses on major themes.
