# Supplementary material for: To neutrally offer or strongly recommend? General practitioners’ perspectives on screening for gestational diabetes according to the national guideline in Norway
Source: Scand J Prim Health Care. 2024 Jul 15;42(4):668–76. doi: 10.1080/02813432.2024.2378204 (PMC11552295; doi:10.1080/02813432.2024.2378204)
Supplement: Appendix 1.pdf [file IPRI_A_2378204_SM3797.pdf]

Appendix 1

Interview guide

Introduction

In April 2017 the Norwegian Directorate of Health published a new national guideline for gestational diabetes. The first chapter of this guideline describe diagnostics and measures for detecting undiagnosed diabetes and gestational diabetes. This includes a recommendation to measure HbA1c in the first trimester for women that fulfills one or more of a specific set of criteria [show or cite the criteria], and a recommendation to conduct a glucose tolerance test in pregnancy week 24-28 for women who fulfill one or more of a specific set of criteria [show or cite the criteria].

Main question:

We would now like to ask you to discuss your experiences with the guideline for gestational diabetes and how you practice screening for undetected diabetes and gestational diabetes as described in the guideline.

Follow-up questions if the answers do not emerge spontaneously:

Do you follow the screening criteria for HbA1c and OGTT as they are stated in the guideline? Or do you adapt these? If so, what are these adaptions based on?

Could you share any experiences of what the screening has led to?
